# Cartilage-protective effects of C-type natriuretic peptide over expression in K/BxN TCR arthritis model

**DOI:** 10.1186/1546-0096-10-S1-A109

**Published:** 2012-07-13

**Authors:** Hulya Bukulmez, Cynthia F Bartels, Kabita Nanda, Tariq M Haqqi, Jean F Welter

**Affiliations:** 1 Case Western Reserve University, Cleveland, OH, USA; 2 MetroHealth Medical Center, Case Western Reserve University, Cleveland, OH, USA; 3 Rainbow Babies and Children, Cleveland, OH, USA; 4 Skeletal Research Center and MetroHealth Medical Center, Case Western Reserve University, Cleveland, OH, USA

## Purpose

The c-type natriuretic peptide (CNP) signaling pathway is known as a major contributor to skeletal growth in children. CNP is produced and secreted by both growth plate and joint chondrocytes, and has a paracrine regulatory effect on cartilage tissue. CNP increases matrix production by chondrocytes and promotes their proliferation. In this study, we investigated whether over-expression of CNP in chondrocytes would be protective of joint cartilage degradation during chronic inflammatory arthritis in vivo.

## Methods

We first developed transgenic mice that over-express CNP (*CNP^col2a1TG^*) in chondrocytes under the control of the collagen 2a1 promoter and enhancer. Then, we obtained K/BxN TCR transgenic mice from a collaborator and analyzed both transgenic mice for their joint cartilage clinical and histologic findings over time. We crossed *CNP^col2a1TG^* mouse with K/BxN TCR transgenic mouse to produce mice with both K/BxN TCR and *CNP^col2a1TG^* backgrounds. The degree of arthritis and cartilage damage in the offspring was analyzed using a clinical scoring system and two histological scoring systems. Differences between the scores were analyzed using the Student’s t-test.

## Results

Mice that carried the transgene for both CNP^col2a1TG^ and K/BxN TCR showed less severe clinical and histologic arthritis findings in the joint cartilage compared to wild type littermates. Between the ages of 6-14 weeks, the average arthritis score of K/BxN TCR transgenic mice that over-expressed CNP was 4.37 ±1.38 (n=8), while the average arthritis score of K/BxN TCR arthritic mice of the same age was 8.66 ±3.26 (n=14), (p<0.05). Histological staining and morphometry did not show any evidence of cartilage degradation in the joint cartilage of CNP^col2a1TG^ mice. The knee and ankle cartilage of CNP^col2a1TG^ mice was thick and showed increased proteoglycan content by Safranin-O staining. However, the double-transgenic offspring mice (K/BxN/CNP^col2a1TG^) developed less cartilage damage and less chondrocyte disorganization while still developing inflammatory changes (pannus) in the synovium, similar to the K/BxN TCR mice. We adapted the ICRS histological scoring system and gave scores to the knee joint cartilage of the 8-week-old male mice. K/BxN mice (n=7) scored significantly lower for both chondrocytic cell distribution (III) and chondrocyte matrix content (II) (p<0.001 and p<0.05, respectively) than the (K/BxN/ CNP^col2a1TG^ mice (n=12).

## Conclusion

K/BxN TCR arthritic mice over-expressing CNP did not have joint cartilage damage due to chronic inflammation. We conclude that excess paracrine production of CNP in the joint cartilage of double-transgenic K/BxN/CNP^col2a1TG^ arthritic mice was able to overcome the effects of pro-inflammatory cytokines on joint cartilage *in vivo*. CNP and the effector molecules of CNP signaling pathway may have therapeutic potential in protecting cartilage homeostasis during chronic inflammatory arthritis.

## Disclosure

Hulya Bukulmez: None; Cynthia F. Bartels: None; Kabita Nanda: None; Tariq M. Haqqi: None; Jean F. Welter: None.

